# Endovascular therapy and medical management of large-core infarcts: prognostic determinants in a retrospective cohort

**DOI:** 10.3389/fneur.2025.1595054

**Published:** 2025-06-03

**Authors:** Yi Li, Hao Tao, Huan Liu, Xiang Fan, Meng-Yu Zhong, Jie Huang, Neng-Wei Yu, Bing-Hu Li

**Affiliations:** ^1^School of Medicine, University of Electronic Science and Technology of China, Chengdu, China; ^2^Department of Neurology, Sichuan Provincial People’s Hospital, Chengdu, China; ^3^School of Clinical Medicine, Southwest Medical University, Luzhou, China

**Keywords:** endovascular therapy, large-core infarcts, prognostic determinants, hyperglycemia, thrombocytosis

## Abstract

**Introduction:**

To investigate the independent prognostic risk factors in acute ischemic stroke patients with large-core infarcts, including patients beyond standard eligibility thresholds for core volume or ASPECTS and provide evidence for early clinical intervention.

**Methods:**

This retrospective cohort study analyzed the clinical data of 96 consecutive patients with large-core infarcts admitted to a regional stroke center between June 2020 and June 2024. Participants were stratified into poor outcome [modified Rankin Scale (mRS) 4–6] and favorable outcome (mRS 0–3) groups based on the 90-day post-intervention assessments. Comparative analyses of the baseline demographics, biochemical parameters, neuroimaging metrics, and treatment modalities were conducted. Univariate analysis followed by multivariate logistic regression was used to identify independent predictors of favorable outcome. A prespecified EVT subgroup analysis was performed, including procedural variables (onset-to-puncture time, puncture-to-recanalization time) and clinical variables in multivariate regression.

**Results:**

Among the 96 patients, 17 (17.7%) achieved favorable outcomes (mRS 0–3) and 79 (82.3%) had poor outcomes (mRS 4–6). Multivariable analysis identified four independent predictors of poor outcome: non-receipt of EVT [OR = 10.22, 95% confidence interval (CI): 1.05–99.76], hyperglycemia (per 1 mmol/L; OR = 1.76, 95% CI: 1.10–2.82), and higher platelet count (per 10^3^/μL; OR = 1.02, 95% CI: 1.00–1.03) (all *p* < 0.05). In the EVT subgroup (*n* = 62), hyperglycemia remained significantly associated with poor outcomes (OR = 1.70, 95% CI: 1.04–2.78, *p* = 0.034).

**Conclusion:**

EVT significantly improves functional outcomes in large-core infarcts. Preoperative hyperglycemia and elevated platelet count are independent predictors of poor outcomes. These findings support standardized protocols that integrate early EVT with glycemic control and coagulation monitoring in this patient population.

## Introduction

According to the Global Burden of Disease Study 2021, stroke remains a leading cause of disability and mortality worldwide, with over 12 million incident cases and 143 million disability-adjusted life-years (DALYs) globally in 2021 (1). Endovascular therapy (EVT) has become a standard approach for patients with acute ischemic stroke (AIS) caused by cerebral large-vessel occlusion (2, 3, 4). Large-core infarcts, typically defined by an Alberta Stroke Program Early CT Score (ASPECTS) ≤5 or an infarct core volume ≥50 mL, are associated with poor outcomes due to extensive ischemic injury. Historically, patients with large cores were excluded from EVT trials due to perceived futility and high hemorrhagic risk. However, several recent randomized controlled trials—the Recovery by Endovascular Salvage for Cerebral Ultra-Acute Embolism-Japan Large Ischemic Core Trial (RESCUE-Japan LIMIT) (5), Endovascular Therapy in Acute Anterior Circulation Large Vessel Occlusive Patients with a Large Infarct Core (ANGEL-ASPECT) (6), Randomized Controlled Trial to Optimize Patient’s Selection for Endovascular Treatment in Acute Ischemic Stroke (SELECT2) (7), Thrombectomy for Emergent Salvage of Large Anterior Circulation Ischemic Stroke (TESLA) (8), The Efficacy and Safety of Thrombectomy in Stroke with Extended Lesion and Extended Time Window (TENSION) (9), and Large Stroke Therapy Evaluation (LASTE) (2)—have demonstrated that EVT plus medical therapy can improve 90-day functional outcomes even in large-core infarcts. These landmark trials herald a new therapeutic paradigm, but real-world prognostic data remain limited. We therefore conducted a retrospective cohort study to identify clinical and imaging determinants of outcome in large-core infarcts patients treated with EVT versus medical management, providing practical insight to guide patient selection and counseling. In addition, controversies persist regarding EVT eligibility for patients with an ASPECTS of 0–2, late presentation (>6 h), or large core volumes (≥100 mL). While subgroup analyses suggest potential benefits for very low ASPECTS, trials such as TESLA failed to meet the primary endpoints in this population, and the outcomes for large cores (>100 mL) remain uncertain (2, 10).

Moreover, although EVTs have significantly improved recanalization rates in patients with LVO, clinical observations reveal that a substantial proportion of individuals undergoing EVT fail to achieve functional independence despite the procedure, with marked heterogeneity in outcomes (11, 12). This paradoxical phenomenon suggests that risk factors of individualized patient beyond procedural success may critically influence prognosis (13). However, current research remains controversial and incomplete, leaving gaps in our understanding of the determinants of post-EVT functional recovery. In recent years, multiple studies have focused on the multidimensional risk factors that influence the prognosis of large-core infarcts. Inflammatory responses and metabolic disturbances (e.g., hyperglycemia and elevated D-dimer levels) have been shown to worsen prognosis by exacerbating oxidative stress and microcirculatory dysfunction (14). However, the role of these biomarkers remains controversial across different treatment modalities, such as EVT and medical management. Current limitations in large-core infarcts prognosis research are primarily reflected in two aspects: first, most studies focus on isolated risk factors, lacking analysis of multidimensional interactions; second, there is limited systematic research on independent risk factors (e.g., preoperative blood glucose and coagulation function) specific to the EVT subgroup, resulting in insufficient evidence-based support for personalized clinical intervention strategies. Emerging evidence suggests that patients with extremely low ASPECTS (≤3) may experience diminished clinical benefits from EVT owing to increased hemorrhagic risk, potentially offsetting therapeutic advantages (15). Conversely, CTP-selected patients with large ischemic cores (>50 mL) might derive functional benefits from EVT, underscoring the necessity of refining imaging thresholds and integrating biological variables (e.g., age and collateral circulation status) into selection protocols (16). Notably, a study focusing on CTP-guided cohorts with baseline core volumes >50 mL demonstrated that while EVT significantly reduced the final infarct volumes in the >70 mL subgroup, functional improvements (assessed via mRS) did not reach statistical significance (11). Furthermore, advanced age (≥75 years) has emerged as a critical prognostic determinant, with all elderly patients exhibiting universally poor outcomes (mRS > 3) at 90 days. These findings underscore the critical importance of incorporating diverse biological determinants, imaging parameters, and patient-specific considerations in optimizing EVT candidate selection in large-core infarcts. This study aimed to identify independent prognostic determinants in patients with AIS and large-core infarcts, specifically focusing on those within the treatment time window eligible for EVT, using multivariable logistic regression with an emphasis on biomarker interactions in the EVT subgroup. Our findings may refine patient selection criteria and optimize therapeutic strategies.

## Methods

This study retrospectively analyzed patients diagnosed with large-core infarcts who received inpatient treatment at Sichuan Provincial People’s Hospital between June 2020 and June 2024. All data were retrieved from the hospital’s electronic medical records.

### Inclusion criteria

Adult patients ≥18 years with AIS, large-core infarcts (ASPECTS ≤5 or ischemic core volume ≥70 mL), within 24 h of onset. Treatment modalities: EVT alone, EVT combined with intravenous thrombolysis (IVT), or medical management. Patients should complete 90-day functional outcome assessment (mRS).

Patients were included for EVT regardless of upper ischemic core volume or lower ASPECTS limits, to explore outcomes in a real-world cohort including patients beyond standard eligibility criteria. Inclusion required either ASPECTS ≤5 or ischemic core volume ≥70 mL.

### Exclusion criteria

Comorbidities included intracranial hemorrhage, pre-stroke mRS ≥2, malignancy, severe organ failure, active infection. Patients with known intracranial vascular malformations were excluded to avoid potential confounding due to altered hemodynamics, hemorrhagic risk, and stroke mechanisms.

### Data collection

Demographics: sex, age, comorbidities (hypertension, diabetes mellitus, and atrial fibrillation), and personal history (tobacco use). Baseline assessments: clinical data including NIHSS score; Neuroimaging data including ASPECTS (ASPECTS scores were independently assessed by experienced neuroradiologists blinded to outcomes; discrepancies were resolved by consensus), CBV index, ischemic core volume (mL; ischemic core was defined using CBV maps with CBV <0.6 mL/100 g threshold; penumbra was defined by mismatch ratio ≥1.8), mismatch ratio; serum biomarkers including glucose, creatinine, BUN, uric acid, PT, fibrinogen, c-reactive protein (CRP), neutrophil count (NEUT), lymphocyte count (LYM), platelet count (PLT), and glycated hemoglobin (HbA1c); treatment modalities data including medical management, EVT, IVT, or combined IVT and EVT; EVT procedural outcomes including reperfusion grade per modified thrombolysis in cerebral infarction (mTICI) criteria and first-pass effect (mTICI 2b/3 in a single attempt), onset-to-recanalization time (ORT, min), puncture-to-recanalization time (PTR, min), onset-to-door time, onset-to-puncture time.

### Outcomes

Functional independence was assessed at 90 days post-intervention using the mRS, which were dichotomized as follows: poor functional outcome: mRS 4–6 (severe disability to death); favorable functional outcome: mRS 0–3 (functional independence to moderate disability).

### Statistical analysis

All statistical analyses were performed using IBM SPSS Statistics software (version 27.0). Continuous variables were compared using the Mann–Whitney *U* test or Student’s *t*-test based on distributional assumptions, whereas categorical variables were analyzed using the *χ*^2^ test or Fisher’s exact test, as appropriate. Multivariate logistic regression was used to evaluate the predictive role of the treatment modalities in functional outcomes. Variables with statistical significance (*p* < 0.05) in the univariate analyses, as well as those with established clinical relevance based on prior studies, were included in the multivariable logistic regression model. In the EVT subgroup, workflow-related variables—including onset-to-puncture time, puncture-to-recanalization time, onset-to-recanalization time, onset-to-door time, and door-to-puncture time—were also subjected to univariate analyses. Selected time metrics were subsequently incorporated into the multivariable analyses to investigate their associations with clinical outcomes. Adjusted odds ratios (OR) with 95% confidence intervals (CI) were calculated, and a two-tailed *p* < 0.05 was considered statistically significant.

## Results

### Patients & baseline characteristics

The study cohort comprised 96 patients, with 17 (17.71%) achieving favorable outcomes (mRS 0–3) and 79 (82.29%) having poor outcomes (mRS 4–6). Intergroup comparisons revealed statistically significant differences in serum glucose levels (*p* = 0.002), platelet counts (*p* = 0.017), and EVT utilization (*p* = 0.005). No significant associations were observed for age, ASPECTS, CBV index, ischemic core volume, penumbra volume, mismatch ratio, NIHSS, BUN, uric acid, PT, fibrinogen, CRP, NEUT, LYM, HbA1c, or demographic/clinical covariates (sex, thrombolysis, smoking history, diabetes mellitus, hypertension, and atrial fibrillation) (all *p* > 0.05) (Table 1).

**Table 1:** Comparison of clinical variables between favorable (mRS 0–3) and poor outcome (mRS 4–6) groups in large-core infarcts.

Variable	Favorable outcome (*n* = 17)	Poor outcome (*n* = 79)	*p*
Age, years median (IQR)	66.00 (54.00, 74.00)	74.00 (64.00, 80.50)	**0.051**
Sex (*n*%)			0.987
Female	37 (46.84)	8 (47.06)	
Male	42 (53.16)	9 (52.94)	
Treatment (*n*%)			
IVT	18 (22.78)	3 (17.65)	0.887
EVT	46 (58.23)	16 (94.12)	**0.005**
Personal history (*n*%)			
Smoking	18 (22.78)	4 (23.53)	1.000
Medical history (*n*%)			
DM	18 (22.78)	2 (11.76)	0.493
HTN	44 (55.70)	6 (35.29)	0.127
AF	56 (70.89)	11 (64.71)	0.615
Imaging index median (IQR)			
ASPECT	3.00 (1.50, 5.00)	2.00 (1.00, 5.00)	0.485
CBVI, %	0.50 (0.40, 0.60)	0.50 (0.30, 0.60)	0.202
Ischemic core, mL	110.50 (85.25, 144.25)	120.50 (91.50, 186.00)	0.238
Ischemic penumbra, mL	121.00 (62.50, 156.00)	84.50 (52.50, 143.00)	0.536
Mismatch ratio, %	2.00 (1.60, 2.70)	1.60 (1.30, 2.25)	0.191
NIHSS	17.00 (12.25, 20.00)	17.00 (12.00, 25.00)	0.486
Laboratory index median (IQR)			
Blood glucose, mmol/L	6.75 (5.55, 7.70)	8.15 (6.78, 9.80)	**0.002**
Cr, μmol/L	64.60 (53.90, 81.80)	71.00 (54.65, 90.42)	0.466
BUN, mmol/L	5.00 (4.02, 6.49)	5.99 (4.92, 7.68)	0.133
UA, μmol/L	345.00 (237.00, 398.00)	338.00 (280.50, 398.00)	0.893
D-dimer, mg/L	1.15 (0.63, 1.28)	2.30 (0.99, 6.22)	**0.020**
PT, s	11.60 (11.10, 11.90)	11.60 (11.20, 12.30)	0.753
FIB, g/L	2.78 (2.24, 3.57)	3.19 (2.49, 3.85)	0.406
CRP, mg/L	2.17 (1.05, 15.40)	7.60 (3.11, 23.48)	0.106
LYM, ×10^9^/L	0.97 (0.76, 1.27)	1.13 (0.76, 1.48)	0.362
NEUT, ×10^9^/L	5.83 (4.62, 8.29)	7.85 (5.86, 10.09)	0.094
PLT, ×10^9^/L	151.00 (112.00, 176.00)	179.00 (140.00, 220.00)	**0.017**
HbA1c, %	5.89 (5.58, 6.14)	6.01 (5.68, 7.04)	0.230

IQR, interquartile range; DM, diabetes mellitus; HTN, hypertension; AF, atrial fibrillation; ASPECT: Alberta Stroke Program Early CT Score; CBV, cerebral blood volume index; NIHSS: National Institutes of Health Stroke Scale; Cr, creatinine; BUN, blood urea nitrogen; UA, uric acid; PT, prothrombin time; FIB, fibrinogen; NEUT, neutrophil count; HbA1c, glycated hemoglobin; PLT, platelet count; LYM, lymphocyte count.

Bold formatting is used to highlight statistically significant results (*p* < 0.05).

### Multivariate regression analysis of predictors of large-core infarcts

**Figure 1:**
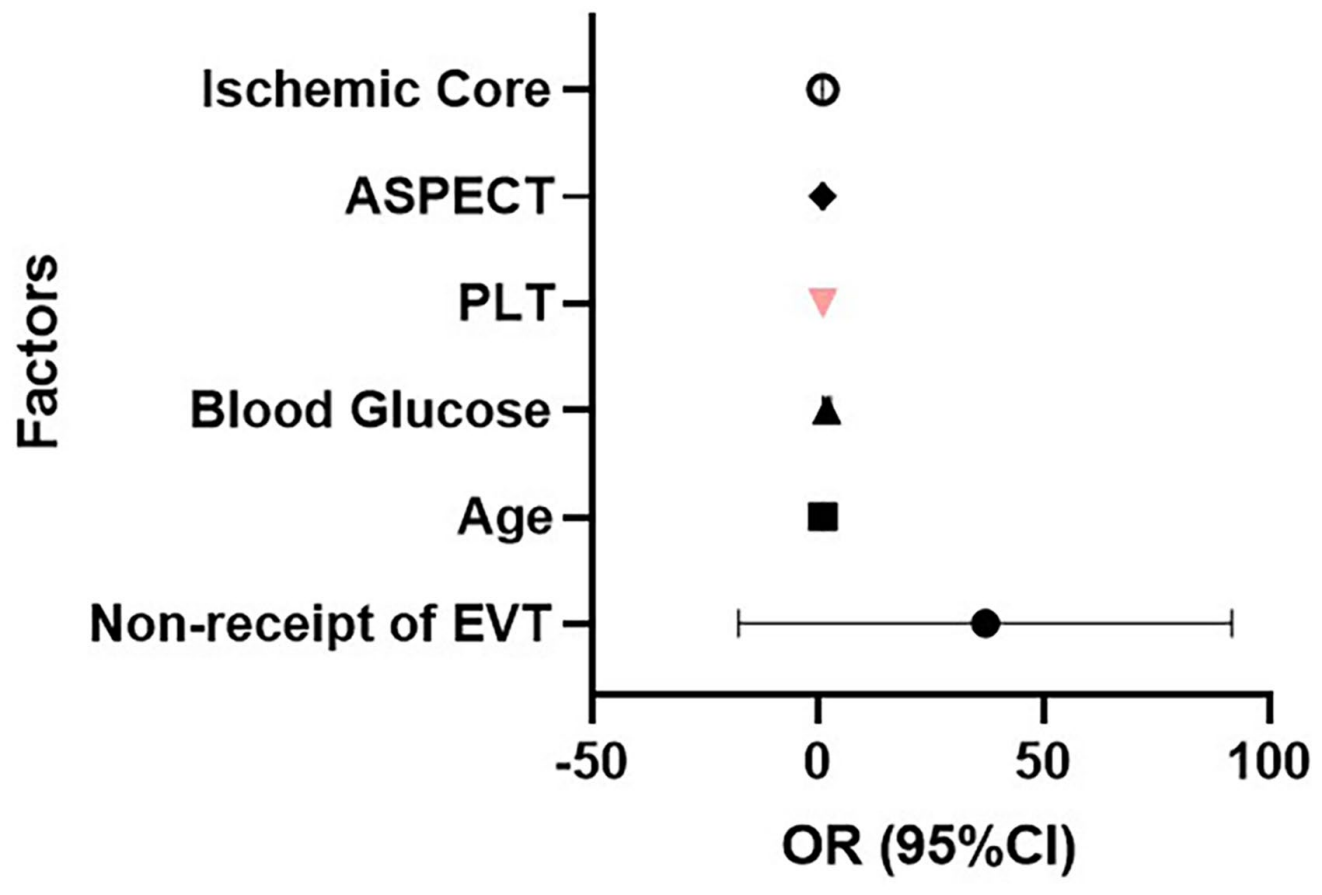
Forest plot of the multivariate logistic regression analysis. Odds ratios (OR) and 95% confidence intervals (CI) for independent predictors of functional outcome are shown on a log scale.

**Figure 2:**
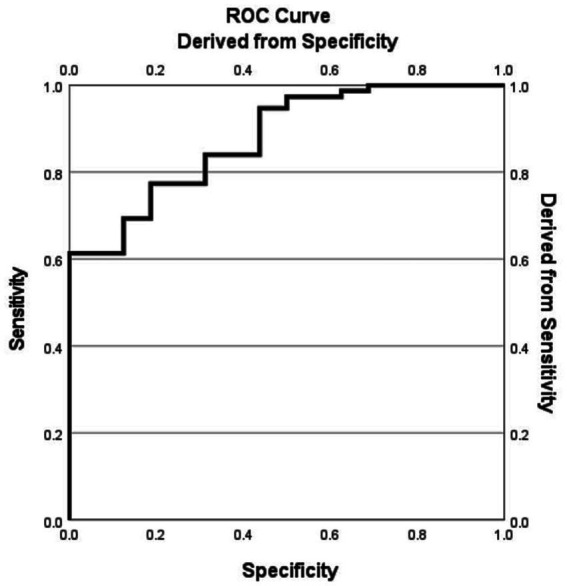
Receiver operating characteristic (ROC) curve of the multivariate logistic regression model predicting functional outcome. The area under the curve (AUC) was 0.877, indicating good discriminative ability.

The functional outcome (favorable: mRS 0–3 vs. poor: mRS 4–6) was set as the dependent variable. Variables showing statistical significance (*p* < 0.05) in the univariate analysis, including non-receipt of EVT, PLT and preoperative blood glucose, were included in the multivariable logistic regression model. In addition, as stated in Method section, established clinical relevance based on prior studies including ischemic core volume, and ASPECT score were also included in the multivariable logistic regression model. Results showed that absence of EVT (adjusted OR = 10.22, 95% CI: 1.05–99.76; *p* = 0.046), elevated blood glucose (adjusted OR = 1.76 per 1 mmol/L increase, 95% CI: 1.10–2.82; *p* = 0.02), and increased platelet count (adjusted OR = 1.02 per 10^9^/L increase, 95% CI: 1.00–1.03; *p* = 0.04) were independently associated with favorable outcome (Table 2 and Figure 1). Furthermore, the receiver operating characteristic (ROC) curve of the multivariate logistic regression model showed an area under the curve (AUC) of 0.887, indicating good discrimination for predicting functional outcome (Figure 2).

**Table 2:** Multivariable logistic regression analysis of factors associated with large-core infarcts.

Variable	*β*	S.E.	Wald (*Z*²)	*p*-value	OR (95% CI)
Non-receipt of EVT	2.32	1.16	4.00	0.046	0.10 (0.10–0.96)
Blood glucose	0.57	0.24	5.51	0.019	0.57 (0.36–0.91)
PLT	0.01	0.010	4.18	0.041	0.99 (0.97–1.00)
Ischemic core	0.01	0.01	2.67	0.102	0.99 (0.98–1.00)
ASPECT	0.03	0.14	0.04	0.847	0.97 (0.74–1.29)
Age	0.02	0.03	0.62	0.430	0.98 (0.93–1.03)

OR, odds ratio; CI, confidence interval.

### Univariate analysis of patients undergoing EVT

Among the 62 patients receiving EVT, 16 (25.81%) achieved favorable outcomes (mRS 0–3), while 46 (74.19%) had poor outcomes (mRS 4–6). Variables with significant differences (*p* < 0.05) were age (*p* = 0.036) and blood glucose levels (*p* < 0.001). Variables without significant differences (*p* > 0.05) were ASPECTS, CBV index, ischemic core volume, penumbra volume, mismatch ratio, NIHSS, creatinine, BUN, UA, PT, FIB, CRP, LYM, NEUT, PLT, HbA1c, sex, first-pass effect, thrombolysis, smoking, hypertension, atrial fibrillation, diabetes mellitus and time metrics (onset-to-door time, onset-to-puncture time, door-to-puncture time, puncture-to-recanalization time, onset-to-recanalization time) (Table 3). Multivariable analysis identified advanced age, elevated blood glucose level, and higher platelet count as independent risk factors for poor prognosis in EVT-treated patients.

**Table 3:** Comparison of clinical variables between favorable and poor outcome groups in patients undergoing EVT (univariate analysis).

Variables	Favorable outcome (*n* = 16)	Poor outcome (*n* = 46)	*p*-value
Age, year, median (IQR)	64.50 (53.50, 74.00)	75.50 (62.25, 80.00)	**0.036**
Sex, *n*%			0.562
Female	7 (43.75)	24 (52.17)	
Male	9 (56.25)	22 (47.83)	
Personal history (*n*%)			
Smoking	4 (25.00)	9 (19.57)	0.918
Medical history (*n*%)			
HTN	6 (37.50)	27 (58.70)	0.143
AF	5 (31.25)	15 (32.61)	0.920
DM	2 (12.50)	10 (21.74)	0.661
FPR	5 (31.25)	12 (26.09)	0.941
Timing metrics, min median (IQR)			
onset-to-arrival time	306.50 (100.75, 511.25)	305.50 (122.25, 490.5)	0.806
onset-to-puncture time	419.00 (259.75, 599.00)	505.00 (293.75, 722.75)	0.337
arrival-to-puncture time	130.50 (82.00, 181.00)	132.00 (86.75, 217.25)	0.636
PTR	50.00 (33.75, 70.50)	54.00 (35.75, 78.75)	0.431
ORT	459.5 (314.50, 665.25)	571.50 (329.25, 805.75)	0.248
Imaging index median (IQR)			
ASPECT	2.50 (1.25, 4.75)	3.00 (1.00, 5.00)	0.958
CBV	0.50 (0.40, 0.60)	0.50 (0.40, 0.60)	0.908
Ischemic core	119.00 (87.50, 148.50)	111.00 (87.00, 184.00)	0.939
Ischemic penumbra	131.50 (71.50, 156.50)	114.00 (71.50, 171.25)	0.902
Mismatch ratio	2.15 (1.55, 2.75)	1.90 (1.55, 2.50)	0.731
NIHSS	17.00 (12.25, 20.00)	17.00 (12.00, 25.00)	0.519
Laboratory index, median (IQR)			
Blood glucose	6.55 (5.51, 7.45)	8.28 (7.02, 9.85)	**<0.001**
Cr	64.80 (55.70, 82.00)	68.00 (53.58, 90.72)	0.898
BUN	5.33 (4.40, 6.67)	6.56 (5.04, 7.63)	0.109
UA	348.00 (261.00, 423.00)	335.00 (278.00, 375.75)	0.647
D-dimer	1.15 (0.59, 1.38)	2.44 (1.05, 5.54)	**0.022**
PT	11.60 (10.95, 12.40)	11.60 (11.33, 12.30)	0.735
FIB	2.77 (2.22, 3.59)	3.10 (2.34, 3.58)	0.754
CRP	2.40 (1.30, 17.86)	5.88 (2.65, 17.65)	0.387
LYM	1.00 (0.73, 1.29)	0.99 (0.74, 1.45)	0.658
NEUT	5.84 (4.85, 8.86)	7.48 (5.68, 9.13)	0.420
PLT	153.00 (117.25, 176.00)	173.00 (139.00, 217.00)	0.068
HbA1c	5.89 (5.46, 6.19)	6.01 (5.69, 6.96)	0.293

PTR: Puncture-to-recanalization time; ORT: Onset-to-Recanalization Time; FPR: First-Pass effect. Bold formatting is used to highlight statistically significant results (*p* < 0.05).

### Multivariable logistic regression in the EVT subgroup

For multivariate logistic regression of the EVT subgroup analysis, covariates were selected based on clinical relevance and statistical significance in univariate analysis. Results showed that higher admission blood glucose level was independently associated with unfavorable outcome (OR = 1.70, 95% CI: 1.04–2.78, *p* = 0.034). Age, onset-to-puncture time, onset-to-door time and puncture-to-recanalization time were not significantly associated with outcome (see Table 4).

**Table 4:** Multivariable logistic regression analysis of factors associated with EVT.

Variables	*β*	S.E.	Wald (*Z*²)	*p*	OR (95% CI)
Age	0.03	0.03	1.72	0.19	1.04 (0.98–1.09)
Onset-to-puncture time	0.00	0.00	0.04	0.85	1.00 (0.99–1.01)
Puncture-to-recanalization time	0.02	0.01	1.16	0.28	1.02 (0.99–1.05)
Onset-to-door time	0.00	0.25	0.12	0.73	1.00 (0.99–1.01)
Blood glucose	0.53	0.37	4.50	**0.03**	1.70 (1.04–2.78)

OR, odds ratio; CI, confidence interval.

Bold formatting is used to highlight statistically significant results (*p* < 0.05).

## Discussion

Large-core infarcts remain a critical challenge in stroke management because of their extensive cerebral involvement and high disability rates. Although EVT has significantly improved outcomes in acute large vessel occlusion (6, 17), data from our center revealed that 74.19% of patients with large-core infarct still experience poor functional outcomes following EVT, underscoring the need to investigate the key determinants of therapeutic efficacy. Our study also identified preoperative hyperglycemia and increased platelet counts as independent risk factors for an unfavorable prognosis in large-core infarcts, providing essential evidence for optimizing perioperative management.

In recent years, EVT indications have expanded to include patients with large-core infarcts (18). Our EVT subgroup achieved a 25.81% rate of favorable outcome (mRS 0–3), which is comparable to the 20–31% reported in recent trials such as SELECT2 and ANGEL-ASPECT (6, 7). The slight differences may be attributable to differences in patient selection criteria, baseline infarct core volumes, and reperfusion success rates. However, heterogeneity in treatment efficacy remains unresolved. Our study showed that among patients with AIS with proximal large-vessel occlusion and a large baseline infarct without an upper size limit, EVT (OR = 10.22) resulted in better functional outcomes and lower mortality than medical therapy alone, aligning with international multicenter trials, such as LASTE. Furthermore, subgroup analysis of EVT-treated patients revealed that preoperative hyperglycemia nearly doubled the risk of unfavorable outcomes (OR = 1.70), suggesting that vascular recanalization alone may be insufficient to counteract secondary injuries triggered by metabolic disturbances. Accumulating evidence indicates that hyperglycemia exacerbates ischemic brain injury through synergistic multi-pathway mechanisms in acute ischemic stroke. It promotes the accumulation of advanced glycation end products (AGEs), which induce vascular endothelial dysfunction and basement membrane thickening, thereby impairing the compensatory capacity of collateral circulation and aggravating ischemic penumbral damage. Concurrently, hyperglycemia triggers sustained neuroinflammatory responses that amplify post-ischemic inflammatory injuries. Furthermore, it induces the overexpression of matrix metalloproteinase-9 (MMP-9), which degrades blood-brain barrier tight junction proteins, compromises barrier integrity, and exacerbates vascular leakage and cerebral edema (19, 20). These interconnected mechanisms create a vicious “hyperglycemia-ischemia injury” cycle, not only expanding infarct core volume and hindering neural repair, but also correlating with poor post-EVT neurological recovery. Although our clinical trial dataset lacks direct evidence of the pathophysiological pathways underlying hyperglycemia-associated adverse outcomes, these findings highlight glycemic control as a potential critical intervention target to improve clinical prognosis.

In addition to hyperglycemia, our study also identified preoperative increased platelet counts as independent risk factors for an unfavorable prognosis in large-core infarcts. Previous study demonstrated that activated platelets release pro-inflammatory cytokines and mediate thrombosis. In patients undergoing EVT, this prothrombotic milieu may predispose patients to post-procedure complications such as early arterial re-occlusion and reperfusion injury, which in turn increase the risk of hemorrhagic transformation (19, 20). It has also been reported platelet counts >300 × 10^9^/L exacerbate microcirculatory dysfunction via enhanced platelet-leukocyte aggregation (14, 21). Intriguingly, Chen et al. recently reported that circulating platelet microparticles (PMPs), but not traditional platelet parameters (e.g., platelet count), were independently correlated with infarct volume (22). This complements our findings that PMPs, as markers of platelet activation, may better reflect thrombogenic activity, whereas elevated platelet counts may amplify ischemic injury through increased blood viscosity and pro-inflammatory mediator release. Collectively, these results underscore the need for a multidimensional platelet function assessment system (e.g., integrating PMPs, platelet count, and aggregation assays) to optimize antithrombotic strategies. These mechanistic insights align with clinical observations that high platelet count independently portend a higher likelihood of futile recanalization and poor functional outcomes despite successful vessel recanalization after EVT.

This study has several limitations: (1) This study is limited by its small sample size, particularly the low number of patients with favorable outcomes (*n* = 17), which constrained the statistical power to detect associations and increased the risk of overfitting in multivariable modeling. Furthermore, the estimates may be imprecise, with wide confidence intervals, and should be interpreted cautiously. Future studies with larger cohorts are warranted to validate these findings. (2) Dynamic monitoring of platelet function markers (e.g., PMPs, P-selectin) was not performed, limiting insight into temporal platelet activation dynamics and their prognostic impact. (3) The EVT subgroup analysis was constrained by limited sample size, necessitating larger cohorts to validate generalizability, and technical EVT details (e.g., number of thrombectomy attempts, stent types) were not analyzed. Future studies should integrate multimodal imaging and molecular biomarkers to develop prognostic prediction models for large core infarcts while exploring the synergistic effects of intensive glycemic control and personalized antiplatelet therapy.

## Conclusion

This study suggests that EVT serves as a protective factor for improving outcomes in large-core infarcts, while preoperative hyperglycemia and increased platelet counts are independently associated with adverse outcomes. Early EVT implementation should be prioritized in clinical practice, coupled with rigorous management of glucose levels and coagulation profiles, to mitigate disability risks. These findings should be interpreted cautiously due to the limited sample size and potential residual confounding. Future multicenter prospective studies are warranted to validate these results and to explore biomarker-guided selection strategies for EVT in large-core infarct patients.

## Data Availability

The original contributions presented in the study are included in the article/supplementary material, further inquiries can be directed to the corresponding authors.
